# Not Baseline Atrial Fibrillation but New-Onset Atrial Fibrillation and the Loss of Left Atrial Function Are Essential for Predicting Poor Outcomes in Non-ischemic Cardiomyopathy

**DOI:** 10.3389/fcvm.2021.781125

**Published:** 2021-12-14

**Authors:** Mana Okune, Masakazu Yasuda, Naoko Soejima, Kazuyoshi Kakehi, Takayuki Kawamura, Takashi Kurita, Gaku Nakazawa, Yoshitaka Iwanaga

**Affiliations:** ^1^Division of Cardiology, Department of Internal Medicine, Faculty of Medicine, Kindai University, Osakasayama, Japan; ^2^Department of Medical and Health Information Management, National Cerebral and Cardiovascular Center, Suita, Japan

**Keywords:** left atrium, new-onset atrial fibrillation, clinical outcomes, non-ischemic cardiomyopathy, magnetic resonance imaging

## Abstract

**Aims:** The clinical impact of the type of atrial fibrillation (AF) has not been completely elucidated in non-ischemic cardiomyopathy (NICM). Although the structure and function of the left atrium (LA) provide prognostic information in patients with heart failure, the relationship of the AF type with LA structure and function in NICM is unclear.

**Methods:** Consecutive patients with NICM who underwent cardiac magnetic resonance were evaluated and followed. Multivariable Cox regression models were used to estimate hazard ratios (HRs) for major adverse cardiovascular events (MACE) related to the AF type, such as paroxysmal AF, chronic AF, and new-onset AF (NOAF).

**Results:** Among 625 patients with NICM (mean age, 64.4 ± 14.2 years; women, 39.7%), 133 had a history of AF at baseline; of these, 60 had paroxysmal AF. Each baseline AF type was associated with higher LA volume and lower LA emptying fraction but not with an increased incidence of MACE (*p* = 0.245). New-onset AF developed in 5.9% of patients with sinus rhythm over a median follow-up period of 609 days, and maximum LA volume was a strong and independent predictor [*p* < 0.001, area under the ROC curve (AUC): 0.795]. Maximum LA volume was superior to LA emptying fraction and B-type natriuretic peptide (AUC: 0.683 and 0.680, respectively). The use of β-blocker and the age of the patient were associated with the incidence of NOAF (HR: 0.37, 95% CI: 0.16–0.84 and HR: 1.05, 95% CI: 1.01–1.09, respectively). Kaplan–Meier analysis showed that patients with NOAF had a higher incidence of MACE than those with sinus rhythm or baseline AF (*p* = 0.002). NOAF and LA emptying fraction were independent predictors of MACE (HR: 2.28, 95% CI: 1.20–3.97 and HR: 0.98, 95% CI: 0.96–0.99, respectively) after adjusting for age, sex, body mass index, and diagnosis.

**Conclusions:** Paroxysmal and chronic AF in patients with NICM were not associated with an increased incidence of MACE despite their association with LA volume and function. NOAF was independently associated with poor prognosis. Higher maximum LA volume predicted the onset and lower LA emptying fraction was independently associated with poor prognosis.

## Introduction

Atrial fibrillation (AF) results in atrial enlargement and is a major arrhythmia associated with heart failure (HF). Hypertension, diabetes, obesity, smoking, and coronary artery disease have been scored as risk factors for AF development (1). AF is classified as paroxysmal or chronic (persistent or permanent) depending on the duration, and among these types, newly developed AF events are attracting attention. A recent study reported that new-onset AF (NOAF) was associated with poor prognosis in acute coronary syndrome (2). Additionally, among patients with HF, NOAF had a significant prognostic impact (3). However, previous studies reported conflicting findings regarding whether baseline AF is an independent predictor of poor prognosis in patients with HF (4). Although NOAF was found to have a significant association with cardiovascular (CV) events in a broad cohort of patients with HF, the clinical impact of NOAF remains unclear in a subcohort of patients with HF, particularly in non-ischemic cardiomyopathy (NICM). NICM, which leads to a decline in cardiac function, results in HF and severe arrhythmic events (5, 6). The relationship of these outcomes with AF types has not been fully elucidated in NICM, and although several factors have been reported, novel predictive factors for the onset of AF need to be explored to prevent the onset of NOAF.

Left atrial (LA) enlargement is a strong predictor of cardiovascular events in the general population and patients with AF or HF (7, 8). A detailed assessment of the structure and function of LA using echocardiography or cardiac magnetic resonance (CMR) provides prognostic information about incident AF, HF onset, and stroke (9, 10). However, the relationship of AF type with LA structure and function in NICM has not been completely examined. In this study, we evaluated the clinical impact of baseline AF and NOAF in patients across a broad spectrum of NICM who underwent CMR examination. We performed a detailed CMR analysis to assess the relationship between LA volume/function and AF type and attempted to elucidate its prognostic potential.

## Methods

### Study Protocol

This was a single-center study in which consecutive patients with cardiomyopathy referred for cardiac magnetic resonance (CMR) were enrolled between February 2008 and December 2018 at Kindai Hospital (Osakasayama, Japan). The referral reason for CMR was to diagnose or evaluate the cardiomyopathy initially, and the patients were in a stable condition. Patients with significant coronary artery disease (significant stenosis of ≥50% of a major coronary artery and history of myocardial infarction) were ruled out by coronary angiography, computed tomography, or CMR (11). NICM was then defined as cardiomyopathy excluding significant primary valve disease, congenital heart disease, acute myocarditis, arrhythmogenic right ventricular cardiomyopathy, and postchemotherapeutic left ventricular dysfunction (12).

The further etiological diagnosis was made based on the following criteria: left ventricular non-compaction (LVNC); the ratio of non-compacted to compacted myocardium in end-diastole of >2.3 by CMR (13); dilated cardiomyopathy (DCM); LV dysfunction and dilatation in the absence of coronary artery disease and specific heart muscle diseases (14); hypertrophic cardiomyopathy (HCM); LV hypertrophy of ≥15 mm and asymmetric/focal hypertrophy in the absence of another disease that could account for the hypertrophy (15); cardiac sarcoidosis (CS); fulfilling the guidelines published in 2016 by the Japanese Circulation Society or the characteristic manifestations and positive findings of echocardiography, ^18^F-fluorodeoxyglucose-positron emission tomography (FDG-PET), or CMR with or without extra CS after exclusion from other known cardiac diseases (16); cardiac amyloidosis (CA); and histological confirmation of amyloidosis by tissue biopsies. The workup at the initial diagnosis included an electrocardiogram (ECG) and echocardiography. The LA diameter and degree of mitral regurgitation (MR) were also assessed. MR was considered to be significant if at least grades 2 of 4 were obtained. The plasma B-type natriuretic peptide (BNP) and serum creatinine levels were measured for clinical purposes. The estimated glomerular filtration rate (eGFR) was calculated using an equation specific to the Japanese population: eGFR = 194 × (serum creatinine) – 1.094 × (age) – 0.287 (× 0.739 for woman).

Paroxysmal AF (PAF) was defined as self-terminating or being cardioverted within 7 days of onset. Chronic AF (CAF), including persistent or permanent AF, lasted >7 days. NOAF was defined if AF was diagnosed or documented during follow-up by the attending physicians among patients without baseline AF (no history of AF and no AF in the baseline ECG).

### CMR Image Acquisition and Analysis

Cardiac magnetic resonance was performed using a 1.5 T scanner (Intera 1.5T; Philips Medical Systems, the Netherlands), according to a standardized protocol. Cine images were acquired with a steady-state free-precession breath-hold sequence (SSFP) in contiguous short-axis slices (10 mm, no gap) from the atrioventricular ring to the apex. Image analysis was performed using commercially available workstations (Aze Virtual Place; Aze, Japan). Endocardial and epicardial contours on short-axis SSFP images were manually delineated on end-diastolic and end-systolic short-axis slices in LV. The summation disk method was used to calculate end-diastolic volume, end-systolic volume, ejection fraction (EF), and masses (17).

To assess LA volume and function, the endocardial borders in two- and four-chamber cine images were contoured carefully, excluding the pulmonary veins and LA appendage. LA volume was calculated based on the biplane area–length method, i.e., LA volume = (0.848 × area_4−ch_ × area_2−ch_)/[(length_2−ch_ + length_4−ch_)/2], based on a previous study (18). Maximum LA volume (Vmax: LA volume at end-systole, immediately before mitral valve opening), minimum LA volume (Vmin: LA volume at end-diastole, immediately before mitral valve closure), and preatrial contraction volume (VpreA: LA volume at the onset of the P-wave on ECG) were defined, and LA emptying fraction [(Vmax–Vmin)/Vmax × 100%], LA passive emptying fraction [(Vmax–VpreA)/Vmax × 100%], and LA active emptying fraction [(VpreA–Vmin)/VpreA × 100%] were calculated (Supplementary Figure 1).

### Clinical Follow-Up

Long-term clinical follow-up, through 2,500 days from CMR testing, was accomplished through a patient-completed questionnaire, telephone interviews, or *via* chart reviews. The following events were recorded: combined major adverse cardiac events (MACE), such as CV mortality, hospitalization for worsening HF, and severe arrhythmia; and all-cause mortality, including the cause of death. CV death was defined as sudden death and death attributed to CV causes, such as fatal myocardial infarction, pump failure, stroke, and severe arrhythmia. Hospitalization for worsening HF was defined as an unexpected presentation to an acute-care facility requiring hospitalization with exacerbation of HF. Severe arrhythmia was defined as sustained ventricular tachycardia/fibrillation, including appropriate implantable cardioverter-defibrillator discharge. All events were based on the clinical diagnosis; however, to validate these events, the medical records were reviewed by us or the physician in charge. They were reviewed independently by two cardiologists (MY and TK), and disagreements were forwarded to a senior cardiologist (YI) for further review.

### Statistical Analysis

The student's *t*-test was performed for comparisons among groups as part of the univariate analysis for continuous variables. Pearson χ^2^ or Fisher's exact test was used to assessing differences in categorical variables using JMP version 14.0 software (SAS Institute, Cary, NC, USA). Furthermore, multiple logistic regression analysis was used to explore the relationship of CMR parameters and BNP level with NOAF. The cutoff levels for NOAF and the sensitivities and specificities of the cutoff levels were calculated using receiver operating characteristic (ROC) curve analysis. Event-free survival curves were analyzed using the Kaplan–Meier method, and the curves were compared using the log-rank test. Univariate and multivariate analyses of the clinical outcomes were evaluated using Cox's proportional hazard model, and hazard ratios (HRs) and 95% CIs were calculated. For incident AF (NOAF), the forward stepwise selection was performed to identify crucial independent variables other than LA Vmax, LA emptying fraction, or log BNP level. For MACE and all-cause mortality, to explore the independent associations between the two variables, NOAF and LV emptying fraction, by univariate analysis, the following covariates were included in the models: age, sex, body mass index, etiological diagnosis, LV EF, and log BNP level after considering collinearity. A value *p* < 0.05 was considered statistically significant. All results were expressed as the mean ± standard deviation.

### Ethical Statement

The study was approved by the Ethics Committee of Kindai University Faculty of Medicine and was conducted according to the principles of the 1964 Declaration of Helsinki and its later amendments. Written informed consent was obtained from all the participants after an explanation of the study's purpose and protocol.

## Results

### Study Population and CMR Findings

In total, 718 consecutive patients with eligible NICM were identified among those with CMR from February 2008 to December 2018. After the exclusion of patients with repeated CMR and poor images, 625 patients with NICM (cohort 1) were enrolled into the analysis, among whom 133 (21.3%) had prevalent AF at baseline. Of these, 73 and 60 patients had CAF and PAF, respectively (Supplementary Figure 2). Table 1 presents the baseline clinical characteristics of cohort 1. The mean age was 64.4 ± 14.2 years, and 39.7% were women. In both the CAF and PAF groups, BNP levels were increased, and the medications, including diuretics and anticoagulants, were administered more frequently than that of sinus rhythm (SR). Only patients with CAF had older age, lower female proportion, lower eGFR, frequent NYHA class ≥ II, and frequent medications, including β-blockers and calcium channel blockers, compared with those with SR. Increased resting HR, increased LA diameter, and frequent moderate or greater MR were also observed only in the patients. The following characteristics were different between the CAF and PAF groups: proportion of women; eGFR; NYHA class ≥ II; medications, including diuretics, anticoagulants, and amiodarone; resting HR; LA diameter; and frequent moderate or greater MR. Regarding the etiological diagnosis of NICM, DCM was most frequently diagnosed in any group, followed by HCM. Patients with DCM had a higher frequency of CAF. Left ventricular ejection fraction was decreased only in the CAF group, and no significant differences were found in other LV parameters among the three groups. Significant differences in LA parameters were found in the three groups. The CAF group showed the largest LA volumes and the lowest LA empty fractions; in contrast, the SR group showed the smallest LA volumes and the highest LA empty fractions.

**Table 1:** Clinical characteristics and CMR findings according to baseline rhythm in cohort 1.

	**Total patients** **(*n* = 625)**	**Sinus rhythm** **(*n* = 492)**	**CAF** **(*n* = 73)**	**PAF** **(*n* = 60)**	***P*** **-value**
Age, years	64.4 ± 14.2	63.1 ± 14.6	70.0 ± 10.0^#^	67.3 ± 12.6	<0.001
Female	248 (39.7%)	204 (41.5%)	19 (25.7%)^#^	25 (41.7%)^†^	0.040
Body mass index, kg/m^2^	23.0 ± 4.3	23.1 ± 4.4	22.8 ± 4.0	22.9 ± 4.1	0.851
eGFR, mL/min/1.73 m^2^	77.5 ± 25.7	79.4 ± 26.0	65.6 ± 18.5^#^	76.7 ± 26.5^†^	<0.001
log BNP	4.7 ± 1.3	4.5 ± 1.4	5.4 ± 0.9^#^	5.3 ± 1.1^#^	0.001
NYHA classification ≥II	246 (39.4%)	170 (34.6%)	48 (65.8%)^#^	28 (46.7%)^†^	<0.001
Etiological diagnosis					0.036
LVNC	51 (8.1%)	46 (9.4%)	1 (1.4%)	4 (6.7%)	
Dilated cardiomyopathy	234 (37.4%)	174 (35.4%)	41 (56.2%)	19 (31.7%)	
Hypertrophic cardiomyopathy	198 (31.7%)	159 (32.3%)	19 (26.0%)	20 (33.3%)	
Cardiac sarcoidosis	124 (19.8%)	100 (20.3%)	12 (16.4%)	12 (20.0%)	
Cardiac amyloidosis	18 (2.9%)	13 (2.6%)	0 (0.0%)	5 (8.3%)	
Medication					
ACE-I/ARB	399 (63.8%)	302 (61.4%)	53 (72.6%)	44 (73.3%)	0.044
β-Blocker	384 (61.4%)	288 (58.5%)	57 (78.1%)^#^	39 (65.0%)	0.004
Calcium channel blocker	176 (28.2%)	127 (25.8%)	28 (38.4%)^#^	21 (35.0%)	0.045
Diuretics	250 (40.0%)	159 (32.3%)	58 (79.5%)^#^	33 (55.0%)^#^^†^	<0.001
MRA	129 (20.6%)	91 (18.5%)	23 (31.5%)^#^	15 (25.0%)	0.033
Anticoagulant	148 (23.7%)	27 (5.5%)	73 (100%)^#^	48 (80.0%)^#^^†^	<0.001
Amiodarone	22 (3.5%)	13 (2.6%)	1 (1.4%)	8 (13.3%)^†^	<0.001
Echo parameters					
Hear rate, beats/min	69.8 ± 15.6	68.3 ± 14.0	79.1 ± 18.9^#^	70.2 ± 18.9^†^	<0.001
Left atrial diameter, mm	41.7 ± 7.4	40.4 ± 6.6	49.6 ± 8.0^#^	42.2 ± 6.5^†^	<0.001
Moderate or greater MR	49 (8.1%)	33 (6.9%)	11 (15.5%)^#^	5 (8.6%)^†^	0.048
CMR parameters					
Left ventricle					
End-diastolic volume, mL	176.0 ± 72.3	175.3 ± 71.7	178.6 ± 69.7	178.2 ± 80.7	0.908
End-systolic volume, mL	115.6 ± 72.9	113.2 ± 72.3	130.2 ± 69.1	117.3 ± 80.8	0.174
Ejection fraction, %	38.7 ± 15.9	40.0 ± 15.7	30.3 ± 15.2^#^	38.8 ± 15.1	<0.001
Mass, g	115.0 ± 44.3	116.3 ± 45.5	108.4 ± 38.4	112.8 ± 40.8	0.335
Left atrium					
Vmax, mL	93.2 ± 44.6	83.5 ± 33.6	143.7 ± 68.0^#^	110.7 ± 41.5^#^^†^	<0.001
Vmax index, mL/m^2^	57.6 ± 27.7	51.5 ± 20.7	88.3 ± 42.6^#^	69.6 ± 26.4^#^^†^	<0.001
Vmin, mL	63.1 ± 41.5	52.3 ± 29.1	121.1 ± 58.3^#^	80.8 ± 39.0^#^^†^	<0.001
Vmin index, mL/m^2^	39.0 ± 25.8	32.3 ± 18.0	74.6 ± 36.7^#^	50.9 ± 24.6^#^^†^	<0.001
Emptying fraction, %	35.9 ± 13.9	39.7 ± 11.9	15.9 ± 5.8^#^	29.1 ± 13.0^#^^†^	<0.001
Passive emptying fraction, %	16.6 ± 7.8	17.5 ± 8.1	12.3 ± 5.2^#^	14.2 ± 6.2^#^^†^	<0.001
Active emptying fraction, %	23.4 ± 13.7	27.0 ± 12.0	4.0 ± 4.1^#^	17.7 ± 12.7^#^^†^	<0.001

*Values are the mean ± SD or n (%).*

# *P < 0.05 vs. sinus rhythm;*

† *P < 0.05 vs. CAF.*

*ACE-I, angiotensin-converting enzyme inhibitor; ARB, angiotensin receptor blocker; BNP, B-type natriuretic peptide; CAF, chronic atrial fibrillation; CMR, cardiac magnetic resonance; eGFR, estimated glomerular filtration rate; LVNC, left ventricle non-compaction; MR, mitral regurgitation; MRA, mineralocorticoid receptor antagonist; NYHA, New York Heart Association; PAF, paroxysmal atrial fibrillation; Vmax, volume at maximum; Vmin, volume at minimum*.

We followed up 492 patients with baseline SR (cohort 2), and 29 (5.9%) patients developed NOAF (NOAF group) over a median follow-up period of 609 days. Table 2 presents the baseline clinical characteristics of the patients in Cohort 2. Significant differences were observed only in BNP levels and LA diameter. Although no significant difference was observed in LV parameters, the NOAF group showed higher LA volumes and lower LA emptying fractions than the SR group. The LA Vmax of the NOAF group was similar to that of the baseline CAF group or PAF group. The LA emptying fraction of the NOAF group was similar to that of the baseline PAF group and higher than that of the baseline CAF group.

**Table 2:** Clinical characteristics and CMR findings according to follow-up rhythm in cohort 2.

	**Sinus rhythm** **(*n* = 463)**	**NOAF** **(*n* = 29)**	***P*** **-value**
Age, years	62.89 ± 14.8	67.4 ± 11.5	0.108
Female	194 (41.9%)	19 (65.6%)	0.432
Body mass index, kg/m^2^	23.0 ± 4.3	23.9 ± 5.3	0.270
eGFR, mL/min/1.73 m^2^	79.5 ± 26.1	77.6 ± 24.8	0.701
log BNP	4.5 ± 1.4	5.4 ± 1.0	0.001
NYHA classification ≥II	155 (33.5%)	15 (51.7%)	0.051
Etiological diagnosis			0.789
LVNC	44 (9.5%)	2 (6.9%)	
Dilated cardiomyopathy	164 (35.4%)	10 (34.5%)	
Hypertrophic cardiomyopathy	147 (31.78%)	12 (41.4%)	
Cardiac sarcoidosis	96 (20.7%)	4 (13.8%)	
Cardiac amyloidosis	12 (2.6%)	1 (3.4%)	
Medication			
ACE-I/ARB	284 (61.3%)	18 (62.1%)	0.938
β-Blocker	272 (58.8%)	16 (55.2%)	0.706
Calcium channel blocker	116 (25.1%)	11 (37.9%)	0.139
Diuretics	147 (31.8%)	12 (41.4%)	0.291
MRA	82 (17.7%)	9 (31.0%)	0.093
Anticoagulant	26 (5.6%)	1 (3.5%)	0.596
Amiodarone	13 (2.8%)	0 (0%)	0.360
Echo parameters			
Heart rate, beats/min	68.56 ± 14.1	64.3 ± 13.1	0.116
Left atrial diameter, mm	40.1 ± 6.5	45.7 ± 7.2	<0.001
Moderate or greater MR	30 (6.7%)	3 (11.1%)	0.381
CMR parameters			
Left ventricle			
End-diastolic volume, mL	173.8 ± 70.3	200.3 ± 88.8	0.053
End-systolic volume, mL	112.1 ± 70.7	130.4 ± 94.5	0.186
Ejection fraction, %	40.0 ± 15.7	39.6 ± 16.8	0.889
Mass, g	115.8 ± 45.4	124.7 ± 48.4	0.305
Left atrium			
Vmax, mL	80.4 ± 29.7	131.9 ± 52.1	<0.001
Vmax index, mL/m^2^	49.7 ± 18.2	80.6 ± 32.9	<0.001
Vmin, mL	49.6 ± 25.2	94.2 ± 49.0	<0.001
Vmin index, mL/m^2^	30.7 ± 15.5	57.8 ± 31.5	<0.001
Emptying fraction, %	40.2 ± 11.7	32.2 ± 12.7	<0.001
Passive emptying fraction, %	17.8 ± 8.9	12.9 ± 5.9	0.001
Active emptying fraction, %	27.3 ± 11.9	22.3 ± 13.1	0.032

*Values are the mean ± SD or n (%).*

*ACE-I, angiotensin-converting enzyme inhibitor; ARB, angiotensin receptor blocker; BNP, B-type natriuretic peptide; CAF, chronic atrial fibrillation; CMR, cardiac magnetic resonance; eGFR, estimated glomerular filtration rate; LVNC, left ventricle non-compaction; MR, mitral regurgitation; MRA, mineralocorticoid receptor antagonist; NYHA, New York Heart Association; PAF, paroxysmal atrial fibrillation; Vmax, volume at maximum; Vmin, volume at minimum*.

Among the cohorts categorized according to the etiological diagnosis of NICM, there was no difference in LA Vmax, but there was a significant difference in the LA emptying fraction (Supplementary Table 1). CA was significantly decreased compared with other diagnoses (*p* < 0.001 for all), and CS was significantly increased compared with DCM (*p* = 0.001). Nevertheless, the prevalence of baseline CAF/PAF and NOAF events did not differ among the etiologies (*p* = 0.081 and 0.805, respectively).

### Clinical Determinants of NOAF

**Figure 1:**
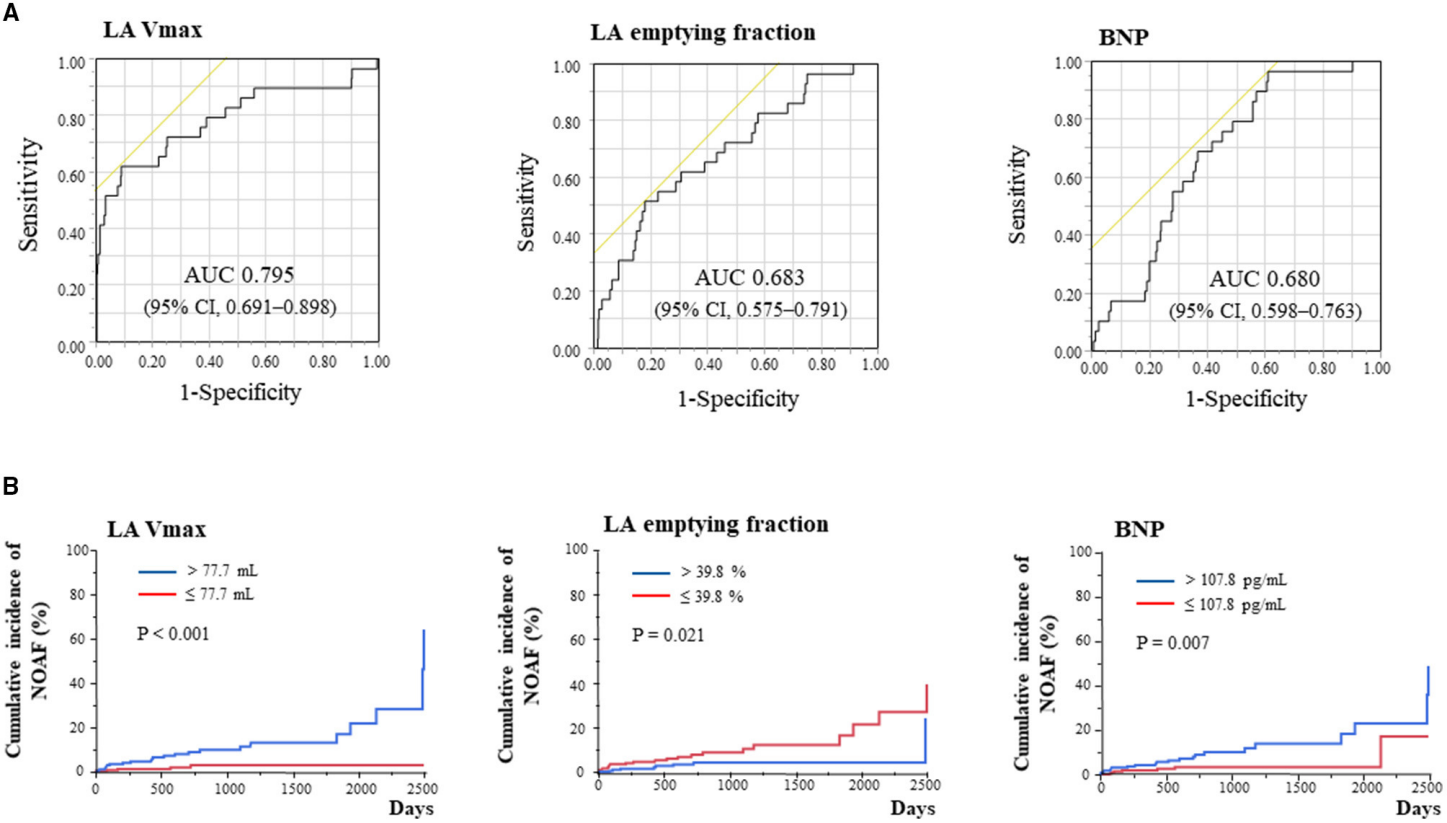
Determinants of new-onset atrial fibrillation (AF) by receiver operating characteristic curve analysis **(A)** and Kaplan–Meier analysis for new-onset AF stratified according to LA Vmax, LA emptying fraction, and BNP **(B)**. AUC, area under the curve; BNP, B-type natriuretic peptide; CI, confidence interval; LA, left atrium; MACE, major adverse cardiac event; NOAF, new-onset atrial fibrillation; Vmax, maximum volume.

Univariate analysis in Cohort 2 showed that age, log BNP levels, LA volumes, LA emptying fraction, and LA passive emptying fraction were associated with the development of NOAF (Supplementary Table 2). In contrast, LV EF and etiological diagnosis did not show an association. The ROC curve analysis showed that LA Vmax showed the best predictive performance of NOAF, with an area under the ROC curve (AUC) of 0.795 (95% CI, 0.691–0.898) (Figure 1A). The optimal cutoff value of LA Vmax was 121.6 ml (sensitivity of 62.1% and specificity of 90.7%). When baseline LA Vmax, LA emptying fraction, or BNP levels were divided by the median value, patients with larger LA Vmax, lower LA emptying fraction, or higher BNP level developed NOAF more in cohort 2 (Figure 1B). Multivariate Cox proportional risk analysis showed that age and β-blocker use in addition to LA Vmax were independent predictors of NOAF (Table 3A). Older patients with higher LA Vmax had a higher risk of developing NOAF than younger patients with lower LA Vmax (odds ratio, 15.64; 95% CI, 2.03–120.30) (Supplementary Figure 3). β-Blocker use was associated with a low incidence of NOAF in the groups with higher LA Vmax (Supplementary Figure 4).

**Table 3:** Multivariate cox proportional hazards analysis.

	**Model 1**		**Model 2**		**Model 3**	
**Variable**	**HR (95% CI)**	***P*** **-value**	**HR (95% CI)**	***P*** **-value**	**HR (95% CI)**	***P*** **-value**
**(A)**						
**For the incidence of NOAF**						
LA Vmax (mL)	1.03 (1.02–1.03)	<0.001				
LA emptying fraction (%)			0.94 (0.91–0.97)	<0.001		
log BNP					1.71 (1.25–2.38)	<0.001
Age (years)	1.03 (1.01–1.09)	0.006	1.03 (1.00–1.06)	0.041	1.03 (1.00–1.06)	0.054
β-Blocker use	0.37 (0.16–0.84)	0.018	0.44 (0.20–0.99)	0.047	0.46 (0.21–1.01)	0.054
**(B)**						
**For MACE**						
NOAF	2.28 (1.20–3.97)	0.014	2.26 (1.19–3.95)	0.015	2.05 (1.08–3.56)	0.029
LA emptying fraction (%)	0.98 (0.96–0.99)	0.002	0.98 (0.96–0.99)	0.005	0.99 (0.87–1.00)	0.139
Age (years)	1.02 (1.00–1.04)	0.027	1.02 (1.01–1.04)	0.007	1.02 (1.00–1.04)	0.014
Sex (male/female)	1.32 (0.88–2.00)	0.183	1.25 (0.84–1.88)	0.279	1.36 (0.91–2.06)	0.129
Body mass index	0.90 (0.85–0.95)	<0.001	0.90 (0.85–0.95)	<0.001	0.92 (0.87–0.97)	<0.001
Diagnosis	—	<0.001				
LV ejection fraction (%)			0.99 (0.98–1.00)	0.163		
log BNP					1.39 (1.15–1.67)	<0.001
**(C)**						
**For all-cause death**						
NOAF	2.68 (1.08–5.70)	0.034	2.71 (1.09–5.85)	0.034	2.59 (1.08–3.73)	0.022
LA emptying fraction (%)	0.99 (0.97–1.02)	0.557	0.99 (0.97–1.01)	0.281	1.00 (0.98–1.03)	0.671
Age (years)	1.04 (1.01–1.08)	0.001	1.05 (1.03–1.09)	<0.001	1.05 (1.02–1.08)	<0.001
Sex (male/female)	1.65 (0.89–3.18)	0.114	1.80 (0.99–3.43)	0.055	1.97 (1.08–3.73)	0.031
Body mass index	0.89 (0.82–0.97)	0.006	0.89 (0.82–0.96)	0.002	0.92 (0.85–1.00)	0.037
Diagnosis	–	<0.001				
LV ejection fraction (%)			1.00 (0.97–1.01)	0.657		
log BNP					1.71 (1.31–2.24)	<0.001

*BNP, B-type natriuretic peptide; CI, confidence interval; HR, hazard ratio, LA, left atrium; LV, left ventricle; MACE, major adverse cardiac events; NOAF, new-onset atrial fibrillation*.

### Clinical Outcomes and the Relationship With LA Geometry/Function

**Figure 2:**
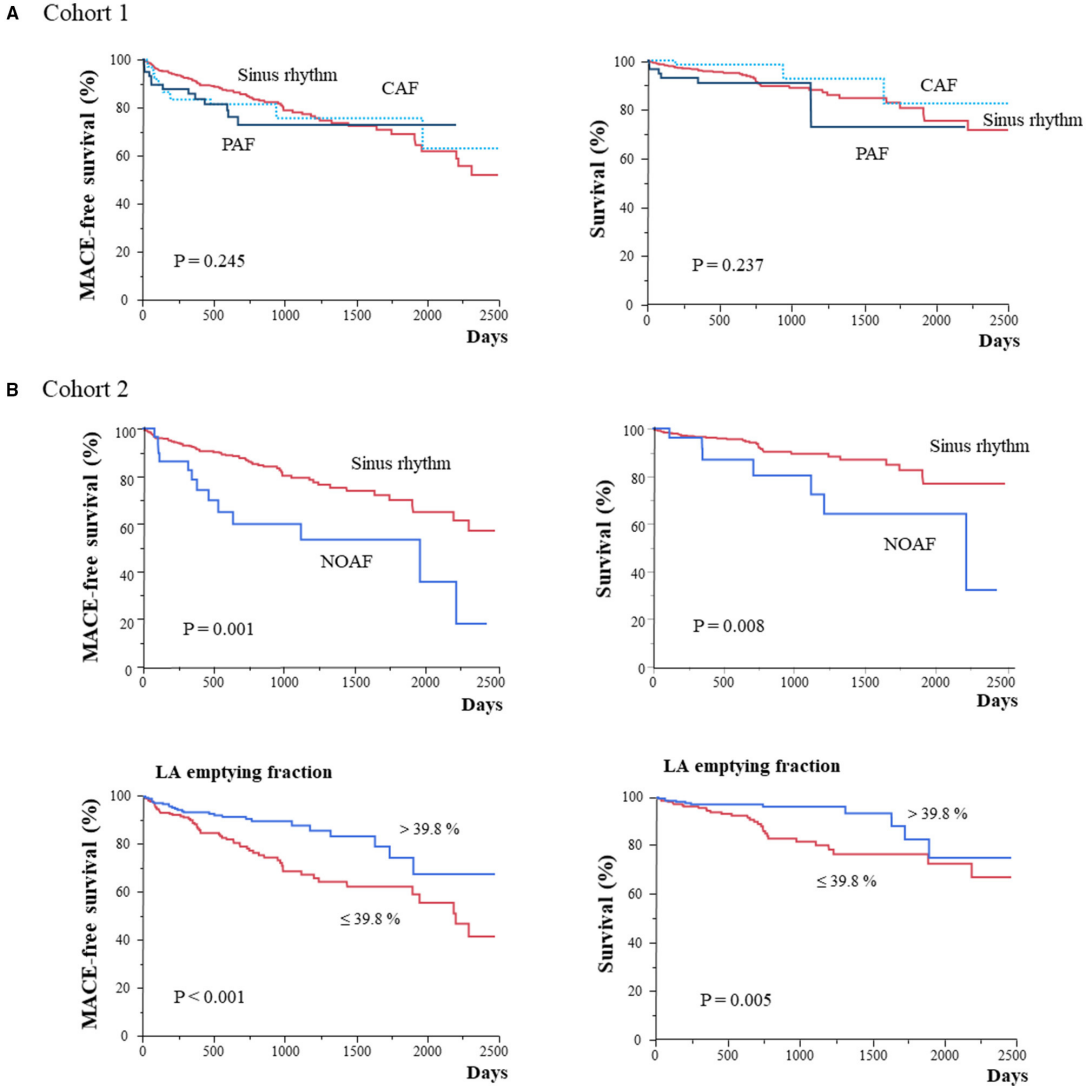
Kaplan–Meier analysis for MACE and all-cause mortality in patients stratified according to AF types or LA function in cohort 1 **(A)** and cohort 2 **(B)**. CAF, chronic atrial fibrillation; LA, left atrium; MACE, major adverse cardiac event; NOAF, new-onset atrial fibrillation; PAF, paroxysmal atrial fibrillation; SR, sinus rhythm.

Kaplan–Meier analysis in cohort 1 revealed no significant differences in both MACE and all-cause death among baseline rhythms (Figure 2A). Each type of AF at baseline was associated with LA volume and function but not with MACE and all-cause death. Patients with baseline CAF and PAF showed more frequent hospitalization for HF (*p* = 0.005; Supplementary Table 3; Supplementary Figure 5A). Conversely, Kaplan–Meier analysis in cohort 2 revealed that NOAF and lower LA emptying fraction (≤ 39.8%) led to a poor prognosis of both MACE and all-cause death (Figure 2B). Moreover, patients with NOAF had MACE, all-cause death, and hospitalization for HF more frequently than those with any type of baseline AF.

Univariate analysis of MACE revealed significant associations of MACE with variables such as age; body mass index; diagnosis; NOAF; NYHA ≥2; log BNP level; and β-blocker, mineralocorticoid receptor antagonist and diuretic use, in clinical characteristics, and LV EF, LA Vmin, and LA emptying fraction in CMR findings (Supplementary Table 2). In the Kaplan–Meier analysis stratified according to diagnosis, only CA showed poor prognosis with a shorter follow-up period (Supplementary Table 1; Supplementary Figure 5B). Multivariate Cox proportional risk analysis revealed that NOAF and LA emptying fraction were independent predictors of MACE (HR, 2.28; 95% CI, 1.20–3.97 and HR, 0.98; 95% CI, 0.96–0.99; respectively) after adjusting for age, sex, body mass index, and the diagnosis (Table 3B). NOAF was also an independent predictor of all-cause death after adjusting for age, sex, body mass index, and diagnosis (HR, 2.68; 95% CI, 1.08–5.70); however, LA emptying fraction was not associated with an all-cause death (Table 3C). LV EF was not associated with both MACE and all-cause death (Model 2), and log BNP was independently associated with both MACE and all-cause death (Model 3).

## Discussion

Atrial fibrillation and HF frequently coexist, and they are closely interrelated, with each disease predisposing to the other (3). In this study, the relationship of AF type with a wide spectrum of NICM, including LVNC, HCM, DCM, CS, and CA, was explored. Patients with prevalent AF had higher age and increased BNP levels and were more symptomatic. Moreover, they took medications such as diuretics and anticoagulants more frequently. Although whether AF is truly independently associated with poor outcomes in HF remains controversial, and this study showed that baseline CAF and PAF were not associated with an increased incidence of MACE and mortality. Similarly, in HCM or CA, AF prevalence has been reported not to be a major contributor to HF morbidity or mortality (19, 20).

In contrast, NOAF (incident AF) was associated with a higher risk of adverse outcomes, which are consistent with findings in other patient groups, including those with acute HF, chronic HF, acute coronary syndrome, and hemodialysis (21–23). In this study, we explored the clinical significance of NOAF in patients across a broad spectrum of NICM, which included a broad spectrum of LV functions. The incidence and prognostic impact of NOAF did not change across LV EF or the diseases groups (systolic or diastolic dysfunction dominant), suggesting that NOAF may be equally crucial in HF with both reduced and preserved EF. Additionally, patients with younger and lower NYHA were included, and they showed a better prognosis than those in previous studies of HF (24, 25). However, the incidence of NOAF was higher than that reported in previous studies. Factors other than HF severity may be associated with the incidence of NOAF. As shown in Tables 2, 3, increased LA volume or decreased LA function may be one of the most important factors. In particular, several events of HF hospitalization soon after the onset of new AF have been reported (24). Furthermore, in this study, MACE after NOAF occurred at a median of 38 days, calculated from the onset of NOAF, and HF hospitalization was the most frequent event among MACE, as shown in Supplementary Table 3. Although its reasons are unknown, NOAF may reflect the instability of HF more generally or it may cause HF admission because of less treatment than other AF types (25).

A unique aspect of this study was the measurement of both BNP levels and LA function/geometry at baseline. This is essential because they are both valuable prognostic markers in HF (10, 25). Several studies demonstrated the association between BNP levels and NOAF in various conditions, including in the general population, in those with acute coronary syndrome, and the postoperative state (26, 27). Although baseline BNP level was a significant predictor of NOAF in this study, the association between them was not strong (AUC: 0.680). LA volume was a better predictor (AUC: 0.795) and was superior to BNP level in patients with NICM (*p* = 0.016). Structural and functional changes in LA have been reported to occur before AF development (9, 28), which this study also suggested in NICM. Recently, besides volumetric analysis, deformational (strain and strain rate) imaging with echocardiography has been used to evaluate LA function. Debonnaire et al. reported that both LA volume and LA strain improved the prediction of NOAF in 242 patients with HCM (29). In addition, the use of β-blocker was negatively associated with the incidence of NOAF in this study. It may decrease the incidence of NOAF through the suppression of the activated sympathetic nervous system (30). It may be useful for further risk stratification to assess novel LA functional imaging, such as deformational (strain and strain rate) imaging or to explore the effect of medications in a cohort of NICM.

Atrial fibrillation progression is often characterized by progression from PAF to CAF. In this study, the deterioration of LA volume and function mirrored this progression; further deterioration of LA volume and function was observed from PAF to CAF. Because PAF or CAF was not associated with MACE or all-cause mortality in this cohort, the deterioration of LA volume and function observed in PAF and CAF did not suggest their direct relationship with the prognosis. However, the LA emptying fraction was associated with a poor prognosis independently of NOAF. A study of HF reported LA dysfunction as a major driver or mediator of clinical decompensation in HF (31). NOAF may be associated with HF decompensation, at least in part, through LA dysfunction; however, how LA dysfunction affects poor prognosis, including HF deterioration, remains unclear. Further studies are necessary to elucidate this for the improvement of clinical management.

This study had several limitations. First, patients across a broad spectrum of NICM at a single center were enrolled; the population may not be completely representative of the entire NICM population. The study population of each diagnosis and the number of patients with NOAF were relatively small, and the total number of events of these patients were also relatively few during follow-up. Any negative findings might be caused by low statistical power. Second, ambulatory serial ECG monitoring might have identified more incident AF than was reported. However, the incidence of NOAF was less or similar to those previously reported (24, 25). While echocardiographic assessment of LA morphology and function is challenging because of the posterior location and thin wall of the LA, CMR has been proposed as the reference method to measure atrial volumes. However, motion artifacts may occur in patients with arrhythmia, such as AF, and data acquisition can be challenging (32). Finally, the study cohort comprised patients who were referred for CMR examination. Thus, the generalizability of our findings might be limited, for example, to patients with severe renal impairment, severe HF, or previously implanted cardiac devices.

## Conclusions

We analyzed 625 consecutive patients across a broad spectrum of NICM who underwent CMR examination, especially in terms of LA geometry and function. Baseline CAF and PAF were well-correlated with the deterioration of LA geometry and function, although they were not associated with an increased incidence of MACE and mortality. Additionally, 5.9% of the patients with baseline SR developed NOAF over a median follow-up of 609 days and were associated with poor prognosis independent of age, sex, body mass index, and diagnosis. LA Vmax showed better predictive performance than LA emptying fraction and BNP. In addition, older age and no β-blocker use were risk factors of NOAF. LA Vmax was not associated with prognosis; rather, the LA emptying fraction was associated with prognosis.

## Data Availability Statement

The raw data supporting the conclusions of this article will be made available by the authors, without undue reservation.

## Ethics Statement

The studies involving human participants were reviewed and approved by Ethics Committee of Kindai University, Faculty of Medicine. The patients/participants provided their written informed consent to participate in this study.

## Author Contributions

MO, MY, and YI: study concept, design, analysis, and interpretation of data. NS, TKa, MO, KK, and MY: data curation. TKu and GN: supervision. MO and MY: writing—original draft. YI: writing—review and editing. All authors contributed to the article and approved the submitted version.

## Funding

This study was supported by grants from the Vehicle Racing Commemorative Foundation (YI).

## Conflict of Interest

The authors declare that the research was conducted in the absence of any commercial or financial relationships that could be construed as a potential conflict of interest.

## Publisher's Note

All claims expressed in this article are solely those of the authors and do not necessarily represent those of their affiliated organizations, or those of the publisher, the editors and the reviewers. Any product that may be evaluated in this article, or claim that may be made by its manufacturer, is not guaranteed or endorsed by the publisher.
