# Supplementary material for: Not Baseline Atrial Fibrillation but New-Onset Atrial Fibrillation and the Loss of Left Atrial Function Are Essential for Predicting Poor Outcomes in Non-ischemic Cardiomyopathy
Source: Front Cardiovasc Med. 2021 Dec 14;8:781125. doi: 10.3389/fcvm.2021.781125 (PMC8712486; doi:10.3389/fcvm.2021.781125)
Supplement: Supplementary file 1 [file Data_Sheet_1.docx]

**Supplementary Figure 1.**

**Measurement of LA volumes by the biplane area–length method**


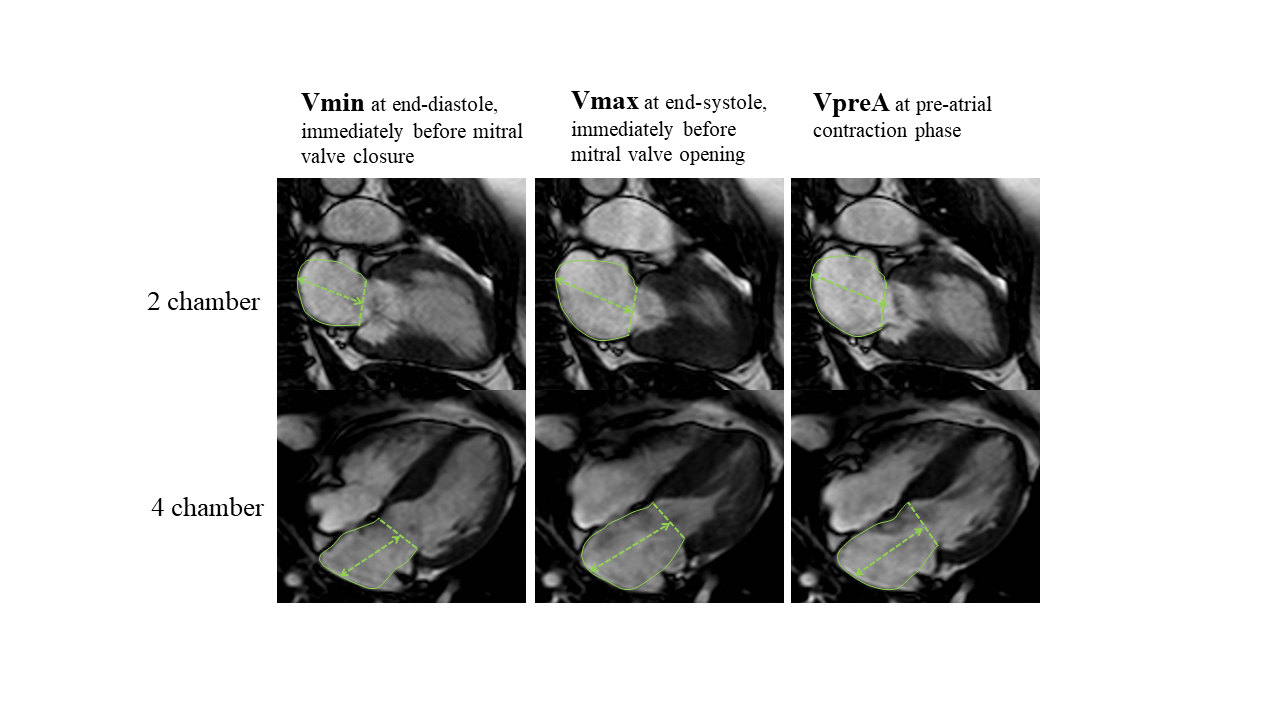


LA, left atrium; Vmax, LA maximum volume at end-systole; Vmin, LA minimum volume at end-diastole; VpreA, pre-atrial contraction volume.

**Supplementary Figure 2.**

**Patient enrollment**

**
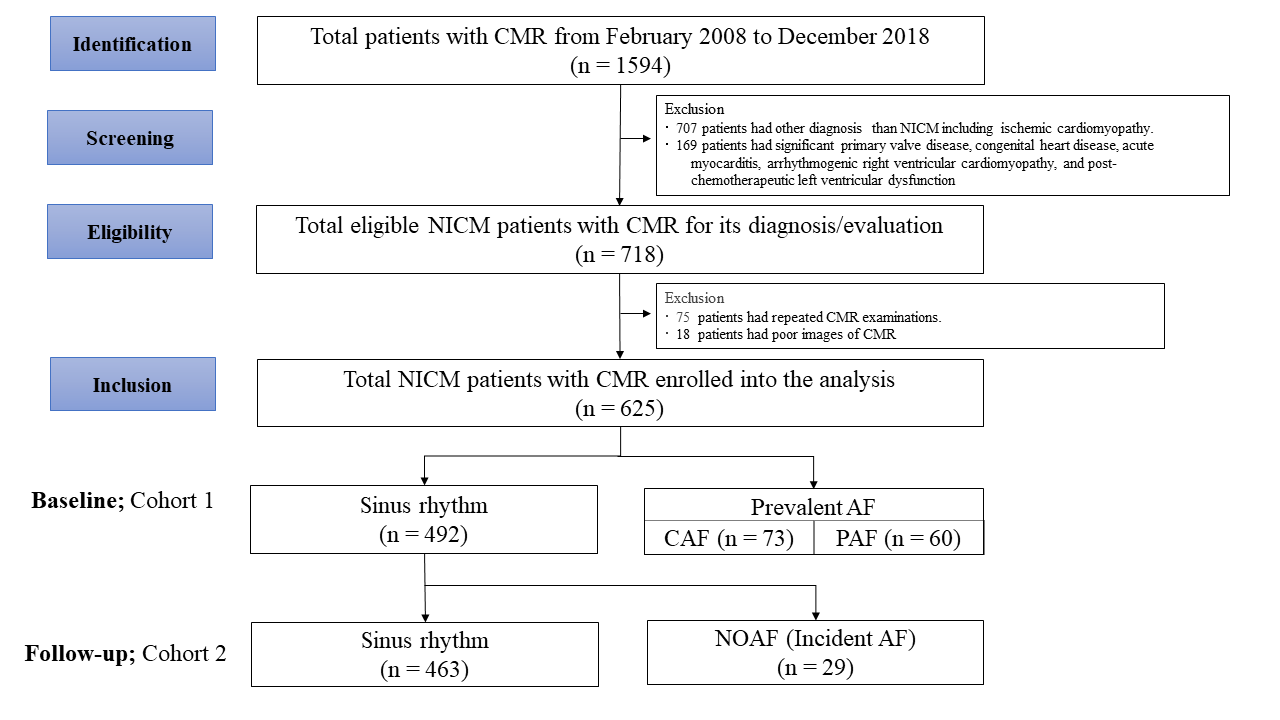
**

AF, atrial fibrillation; CAF, chronic atrial fibrillation; CMR, cardiac magnetic resonance; NICM, nonischemic cardiomyopathy; NOAF, new-onset atrial fibrillation; PAF, paroxysmal atrial fibrillation.

**Supplementary Figure 3.**

**Risk stratification of incidence of NOAF by age and LA volume**


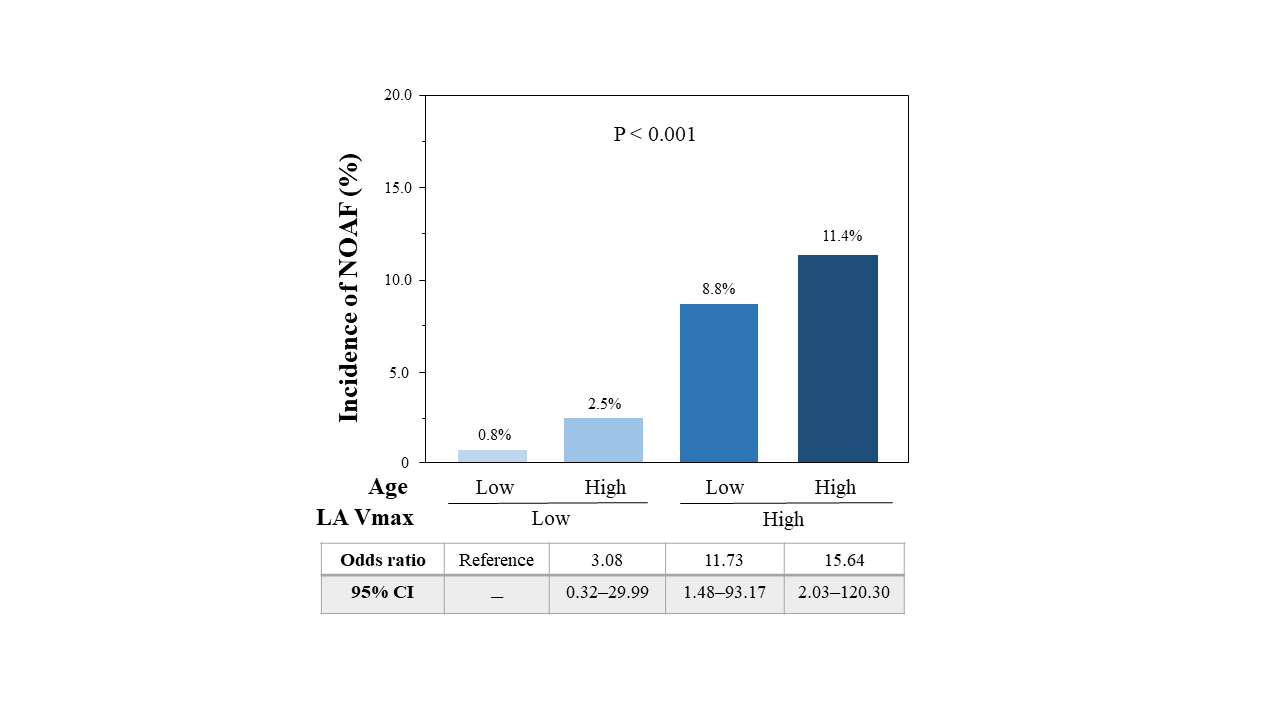


The patients were divided into 4 groups by a median value of age or LA V max (66 years and 77.7 mL, respectively).

CI, confidence interval; LA Vmax, left atrial maximum volume; NOAF, new-onset atrial fibrillation.

**Supplementary Figure 4.**

**Risk stratification of incidence of NOAF by β-blocker use in addition to age and LA volume**


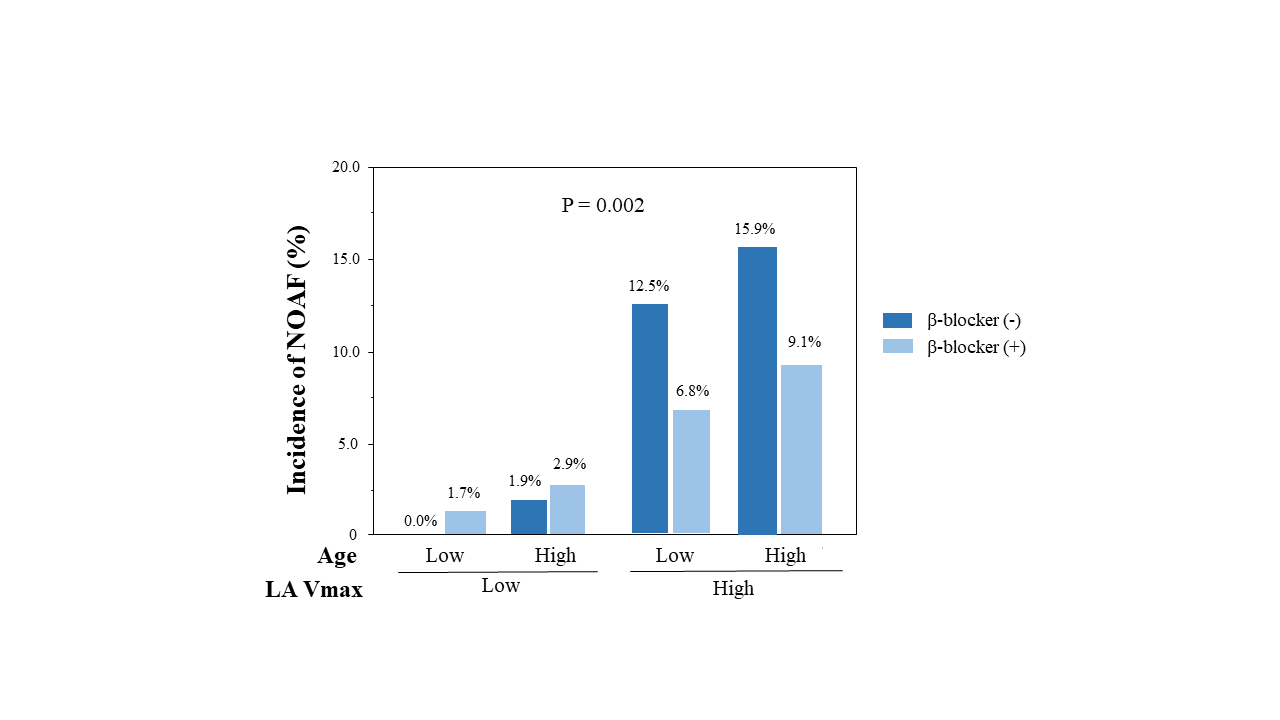


The patients were further divided by β-blocker use in addition to age and LA V max grouping.

LA Vmax, left atrial maximum volume; NOAF, new-onset atrial fibrillation.

**Supplementary Figure 5.**

**Kaplan–Meier analysis for hospitalization for HF, MACE, or all-cause mortality in patients stratified according to baseline AF types (A) or diagnosis (B) in Cohort 1**


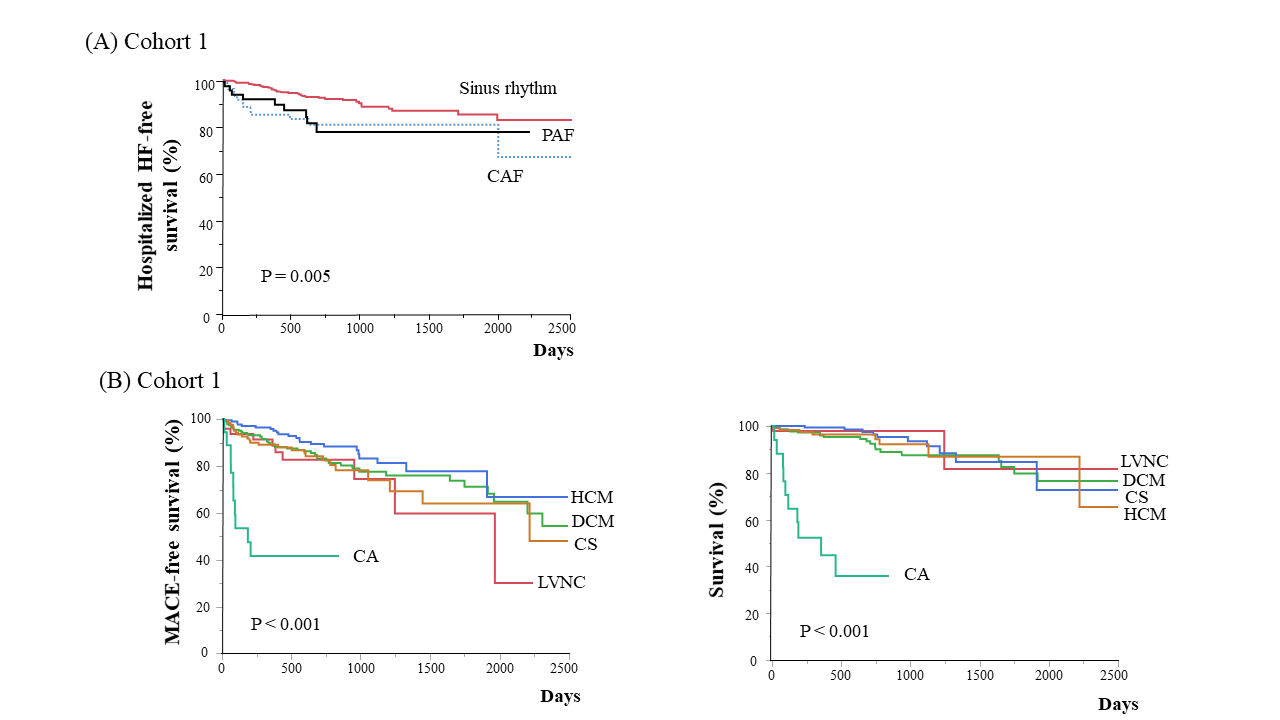


CA, cardiac amyloidosis; CAF, chronic atrial fibrillation; CS, cardiac sarcoidosis; DCM, dilated cardiomyopathy; HCM, hypertrophic cardiomyopathy; HF, heart failure; LVNC, left ventricle non-compaction; MACE, major adverse cardiac events; PAF, paroxysmal atrial fibrillation

**Supplementary Table 1. Clinical characteristics, CMR findings, and outcomes according to the diagnosis in Cohort 1**

|  | Total  (n = 625) | LVNC  (n = 51) | DCM  (n = 234) | HCM  (n = 198) | CS  (n = 124) | CA  (n = 18) | P value |
| --- | --- | --- | --- | --- | --- | --- | --- |
| Age, years | 64.4 ± 14.2 | 63.8 ± 14.3 | 62.1 ± 15.0 | 65.8 ± 14.4 | 65.3 ± 11.9 | 72.3± 8.0 | 0.006 |
| Female | 248 (39.7%) | 20 (39.2%) | 78 (33.3%) | 83 (41.9%) | 60 (48.4%) | 7 (38.9%) | 0.081 |
| log BNP | 4.7 ± 1.3 | 4.7 ± 1.5 | 4.8 ± 1.4 | 4.8 ± 1.2 | 4.4 ± 1.4 | 6.1 ± 0.9 | <0.001 |
| CAF/PAF | 133 (21.3%) | 5 (9.8%) | 60 (25.6%) | 39 (19.7%) | 24 (19.3%) | 5 (27.8%) | 0.081 |
| NOAF | 29 (6%) | 2 (4%) | 10 (4%) | 12 (6%) | 4 (3%) | 1 (6%) | 0.805 |
| AUC of LA Vmax | 0.795 | – | 0.836 | 0.801 | 0.938 | – |  |
| CMR data |  |  |  |  |  |  |  |
| LV EF, % | 38.7 ± 15.9 | 28.0 ± 11.9 | 26.8 ± 11.1 | 51.5 ± 9.8 | 44.1 ± 14.3 | 45.5 ± 10.0 | <0.001 |
| LA Vmax, mL | 93.2 ± 44.6 | 94.0 ± 44.4 | 95.5 ± 42.1 | 96.5 ± 51.9 | 83.3 ± 38.0 | 90.9 ± 19.8 | 0.096 |
| LA emptying fraction, % | 35.9 ± 13.9 | 36.1 ± 13.6 | 34.0 ± 14.1 | 37.1 ± 12.5 | 39.8 ± 14.5 | 20.9 ± 9.3 | <0.001 |
| Follow-up outcomes |  |  |  |  |  |  |  |
| Period,  median days (IQR) | 610  (245, 927) | 459  (235, 812) | 664  (287, 948) | 647  (233, 987) | 610  (295, 838) | 141  (63, 421) | 0.002 |
| MACE | 109 (17%) | 10 (20%) | 43 (18%) | 22 (11%) | 24 (19%) | 10 (56%) | <0.001 |
| All-cause death | 51 (8%) | 2 (4%) | 20 (9%) | 10 (5%) | 8 (6%) | 11 (61%) | <0.001 |

Values are the mean ± SD or number (%).

AUC, area under the curve; BNP, B-type natriuretic peptide; CA, cardiac amyloidosis; CAF, chronic atrial fibrillation; CMR. Cardiac magnetic resonance; CS, cardiac sarcoidosis; DCM, dilated cardiomyopathy; EF, ejection fraction; HCM, hypertrophic cardiomyopathy; IQR, interquartile range; LA, left atrium; LV, left ventricle; LVNC, left ventricle non-compaction; MACE, major adverse cardiac events; NOAF, new-onset atrial fibrillation; PAF, paroxysmal atrial fibrillation; Vmax, volume at maximum.

**Supplementary Table 2. Univariate Cox regression analysis for NOAF and MACE**

|  | NOAF (Cohort 2) | | MACE (Cohort 1) | |
| --- | --- | --- | --- | --- |
| **Variables** | **HR (95% CI)** | **P value** | **HR (95% CI)** | **P value** |
| **Age (years)** | 1.03 (1.00–1.07) | 0.022 | 1.04 (1.02–1.05) | <0.001 |
| **Sex (male/female)** | 1.33 (0.63–2.98) | 0.462 | 1.02 (0.70–1.52) | 0.915 |
| **Body mass index (kg/m^2^)** | 1.02 (0.94–1.10) | 0.626 | 0.88 (0.84–0.93) | <0.001 |
| **Diagnosis** | – | 0.485 | – | <0.001 |
| **CAF/PAF** | – | – | 1.37 (0.87–2.09) | 0.167 |
| **NOAF** | – | – | 2.38 (1.27–4.10) | 0.009 |
| **NYHA ≥2** | 1.62 (0.77–3.43) | 0.199 | 3.38 (2.25–5.21) | <0.001 |
| **eGFR (mL/min/1.73 m²)** | 0.99 (0.98–1.01) | 0.354 | 0.99 (0.99–1.00) | 0.150 |
| **log BNP** | 1.60 (1.20–2.17) | 0.001 | 1.63 (1.39–1.91) | <0.001 |
| **ACE-I/ARB** | 0.99 (0.47–2.16) | 0.973 | 1.29 (0.86–1.97) | 0.222 |
| **β-Blocker** | 0.77 (0.37–1.63) | 0.482 | 1.55 (1.03–2.40) | 0.035 |
| **MRA** | 2.22 (0.96–4.75) | 0.062 | 1.62 (1.06–2.42) | 0.028 |
| **Calcium channel blocker** | 2.00 (0.91–4.24) | 0.084 | 0.88 (0.56–1.33) | 0.540 |
| **Diuretics** | 1.34 (0.62–2.79) | 0.444 | 2.44 (1.66–3.62) | <0.001 |
| **Anticoagulant** | 0.69 (0.04–3.23) | 0.695 | 1.41 (0.92–2.12) | 0.114 |
| Heart rate (beats/min) | 0.97 (0.95–1.00) | 0.055 | 1.00 (0.99–1.01) | 0.943 |
| LA diameter (mm) | 1.12 (1.06–1.17) | <0.001 | 1.02 (0.99–1.04) | 0.216 |
| Moderate or greater MR | 1.03 (0.24–3.00) | 0.962 | 0.78 (0.36–1.46) | 0.460 |
| LV EF (%) | 1.02 (0.98–1.03) | 0.552 | 0.98 (0.97–0.99) | 0.003 |
| LV EDV (mL) | 1.00 (1.00–1.01) | 0.257 | 1.00 (1.00–1.00) | 0.544 |
| LV ESV (mL) | 1.00 (1.00–1.01) | 0.596 | 1.00 (1.00–1.00) | 0.163 |
| LV mass (g) | 1.00 (0.99–1.01) | 0.658 | 1.00 (0.99–1.00) | 0.183 |
| LA Vmax (mL) | 1.02 (1.02–1.03) | <0.001 | 1.00 (1.00–1.01) | 0.188 |
| LA Vmin (mL) | 1.02 (1.02–1.03) | <0.001 | 1.00 (1.00–1.01) | 0.023 |
| LA emptying fraction (%) | 0.95 (0.92–0.98) | 0.001 | 0.97 (0.95–0.98) | <0.001 |
| LA passive emptying fraction (%) | 0.90 (0.84–0.95) | <0.001 | 0.94 (0.91–0.96) | <0.001 |
| LA active emptying fraction (%) | 0.97 (0.94–1.00) | 0.079 | 0.98 (0.96–0.99) | 0.002 |

ACE-I, angiotensin-converting enzyme inhibitor; ARB, angiotensin receptor blocker; BNP, B-type natriuretic peptide; CAF, chronic atrial fibrillation; CI, confidence interval; CMR, cardiac magnetic resonance; EDV, end-diastolic volume; EF, ejection fraction; eGFR, estimated glomerular filtration rate; ESV, end-systolic volume; HR, hazard ratio; LA, left atrium; LV, left ventricle; MR, mitral regurgitation; MRA, mineralocorticoid receptor antagonist; NYHA, New York Heart Association; PAF, paroxysmal atrial fibrillation; Vmax, volume at maximum; Vmin, Volume at minimum

**Supplementary Table 3. Clinical events according to types of AF**

| Cohort 1 | Total (n=625) | Sinus rhythm (n=492) | CAF (n=73) | PAF (n=60) | P value |
| --- | --- | --- | --- | --- | --- |
| **MACE** | 109 (17.4%) | 82 (16.7%) | 14 (19.2%) | 13 (21.7%) | 0.576 |
| **CV death** | 29 (4.6%) | 25 (5.1%) | 2 (2.7%) | 2 (3.3%) | 0.889 |
| **Hospitalization for HF** | 54 (8.6%) | 35 (7.1%) | 10 (13.7%) | 9 (15.0%) | 0.013 |
| **Severe arrhythmia** | 26 (4.2%) | 22 (4.5%) | 2 (2.7%) | 2 (3.3%) | 0.205 |
| **All-cause death** | 51 (8.2%) | 42 (8.5%) | 3 (4.1%) | 6 (10.0%) | 0.375 |
| **ICD/CRT implantation** | 44 (7.0%) | 8 (1.6%) | 1 (1.4%) | 8 (13.3%) | 0.043 |

| Cohort 2 | Total (n=492) | Sinus rhythm (n=463) | NOAF (n=29) | P value |
| --- | --- | --- | --- | --- |
| **MACE** | 82 (16.7%) | 69 (14.9%) | 13 (44.8%) | <0.001 |
| **CV death** | 25 (5.1%) | 23 (5.0%) | 2 (6.9%) | 0.289 |
| **Hospitalization for HF** | 35 (7.1%) | 28 (6.0%) | 7 (24.1%) | <0.001 |
| **Severe arrhythmia** | 22 (4.5%) | 18 (3.9%) | 4 (13.8%) | 0.045 |
| **All-cause death** | 42 (8.5%) | 35 (7.6%) | 7 (24.1%) | 0.002 |
| **ICD/CRT implantation** | 8 (1.6%) | 7 (1.5%) | 1 (3.5%) | 0.640 |

AF, atrial fibrillation; CAF, chronic atrial fibrillation; CRT, cardiac resynchronization therapy; CV, cardiovascular; ICD, implantable cardioverter defibrillator; HF, heart failure; MACE, major adverse cardiac events; NOAF, new-onset atrial fibrillation; PAF, paroxysmal atrial fibrillation
